# 

**Published:** 1897-02

**Authors:** 


### CONTENTS.

ORIGINAL ARTICLES:
The Cattle-tick Plague: Preventive Treatment. By COOPER CURTICE, Veterinarian . . . . . 67
Abortion in Domesticated Animals. By CHARLES F. DAWSON . . . . . 75
Bacteriology in its Relation to Veterinary Science. By M. P. RAVENAL, M.D. . 79
Acetanilid (or Antifebrin). By F. S. ALLEN, M.D., D.V.S. . . . . . 84
Communicable Animal Diseases. By W. HERBERT LOWE, D.V.S. . . . . 87
ABSTRACTS FROM FOREIGN JOURNALS:
The Propagation of Cattle Tuberculosis by Fecal Matter—Contribution to the Study of the Horse-chestnut—Chlorobarium—Length of Pace in Horses—Turpentine in Colic—Prof. Hobday's Results in Median Nenrectomy—Horse-disease on the Kankakee River . . . . . 90
REPORTS OF CASES:
Necrosis Totalis of the Scapula. By J. B. NIGHBERT, V.S. . . . . . 95
Treatment of Anasarca by the Subcutaneous Injection of Antistreptococcus Serum (Marmorek). By HAROLD SORBY . . . . . 97
Outward Luxation of the Femur. By W. E. A. WYMAN, V.S. . . . . . 97
EDITORIALS:
New York State Legislation—Association Work in Virginia and Chicago—Unprofessional Conduct in a Member of the U. S. V. M. A.—Marriage—Necrology 98–100
LEGISLATION:
Pennsylvania—Massachusetts—Indiana—New York—Montana . . . . . 100–102
CONTROL WORK:
Pennsylvania—Iowa—Oregon—National . . . . . 102–104
SOCIETY PROCEEDINGS:
Veterinary Medical Association of New York County—Western Ontario Veterinary Medical Association—Maine Veterinary Medical Association—Ontario Veterinary Association—Montreal Veterinary Medical Association—Virginia State Veterinary Medical Association—Wisconsin Society of Veterinary Graduates—Pennsylvania State Veterinary Medical Association—Niagara and Orleans County Veterinary Medical Society—Veterinary Medical Society of the University of Pennsylvania—Chicago Veterinary Society . . . . . 105–114
PERSONAL—FACTS AND FANCIES—GLEANINGS . . . . . 115–120

